# Imagery datasets for photobiological lighting analysis of architectural models with shading panels

**DOI:** 10.1016/j.dib.2022.108278

**Published:** 2022-05-15

**Authors:** Mojtaba Parsaee, Claude MH Demers, Marc Hébert, Jean-François Lalonde

**Affiliations:** a GRAP (Groupe de Recherche en Ambiances Physiques), School of Architecture, Laval University, Quebec, Canada; b CERVO Brain Research Centre, Faculty of Medicine, Laval University, Quebec, Canada; c Computer Vision and Systems Lab, Department of Electrical and Computer Engineering, Laval University, Quebec, Canada

**Keywords:** High and low dynamic range image, Daylight, Surface, Color, Adaptive façade, Interior design, Healthy building, Computer vision

## Abstract

This paper describes eight imagery datasets including around 12000 images grouped in 1220 sets. The images were captured inside an architectural model aimed at exploring the impact of shading panels on photobiological lighting parameters. The architectural model represents a generic space at 1:10 scale with a single side fully glazing façade used to install shading panels. The datasets present interior lighting conditions under different shading configurations in terms of surface colors and glossiness, horizontal and vertical orientations and upwards, downwards, and left/right inclinations of panels, V-shape opening, low to high densities, and top and bottom positions at the window. The experiments of shading panel configurations were conducted under four to six different exterior overcast daylighting conditions simulated with very cool to very warm color temperatures and high to low intensities inside an artificial sky chamber. The datasets include bracketed low dynamic range (LDR) images which enable generating high dynamic range (HDR) images for photobiological lighting evaluations. Images were captured from the side and back viewpoints inside the model by using Raspberry Pi camera modules mounted with fisheye lenses. The datasets are reusable and useful for architects, lighting designers, and building engineers to study the impact of architectural variables and shading panels on photobiological lighting conditions in space. The datasets will also be interesting for computer vision specialists to run machine learning techniques and train artificial intelligence for architectural applications. The datasets are partially used in Parsaee, et al. [1]. The datasets are compiled as part of a doctoral dissertation in architecture at Laval University authored by Mojtaba Parsaee [2]. The datasets are shared through two Mendeley data repositories [3,4].

## Specifications Table

**Table:** 

Subject	Architecture
Specific subject area	The datasets contribute to healthy building developments, and daylighting, façade, interior and color design in architecture.
Type of data	Table Image Figure
How the data were acquired	Bracketed low dynamic range (LDR) images were captured from side and back viewpoints inside the 1:10 scale architectural model using Raspberry Pi camera modules mounted with fisheye lenses. The architectural model is created based on Jafarian, et al. [5,6], Poirier, et al. [7], Ruck, et al. [8], Baker, et al. [9]. The camera is manufactured as RPi Camera-I by WaveShare [10]. This model has a fixed aperture value of f/2, a focal length of 1.55mm, and a 5-megapixel OV5647 sensor with a CCD size of 1/4inch which could produce images with 2592-by-1944-pixel resolutions. The fisheye lens has a diagonal angle of view of around 185-degree. The camera and fisheye lens specifications are fully described in WaveShare [10]. Multiple LDR images were photographed from very dark to very bright high exposure values (i.e., -2 to +3 EVs) provided by modifying the camera shutter speeds from around 15 to 1/2 seconds. All LDR images were captured with an ISO-100 and a fixed white balance (D65). A Python script using OpenCV libraries and ExifTool is developed to generate HDR images from LDR images. The HDR images were calibrated for photobiological lighting analysis based on Jung [11], Jung and Inanici [12].
Data format	Raw Analyzed
Description of data collection	Eight imagery datasets were captured inside a 1:10-scale architectural model with 23 external shading panels tested under diffuse artificial skies with six lighting conditions offering very cool to very warm correlated color temperatures (CCT) with high to low intensities.
Data source location	Institution: Laval University City, Province: Quebec, Quebec Country: Canada Latitude and longitude: [46° 48′ N, 71° 12′ W]
Data accessibility	Two Mendeley Data repositories are created to share the datasets as the Mendeley Data offer maximum 10 GB space per data repository. 1. Imagery datasets for photobiological lighting analysis of architectural models with colored shadings (No. 1) Data identification number: doi:10.17632/j4zzgfy8gy.2 Direct URL to data: https://data.mendeley.com/datasets/j4zzgfy8gy/3 2. Imagery datasets for photobiological lighting analysis of architectural models with colored shadings (No. 2) Data identification number: DOI: 10.17632/7tv8yb5647.2 Direct URL to data: https://data.mendeley.com/datasets/7tv8yb5647/3
Related research article	The datasets are partially used in the following paper. M. Parsaee, C. M. H. Demers, A. Potvin, J.-F. Lalonde, M. Inanici, and M. Hébert, "Biophilic photobiological adaptive envelopes for sub-Arctic buildings: Exploring impacts of window sizes and shading panels’ color, reflectance, and configuration," *Solar Energy,* vol. 220, pp. 802-827, 2021. https://doi.org/10.1016/j.solener.2021.03.065

## Value of the Data

• These imagery datasets can be used to evaluate the impact of external shading panel characteristics on photobiological lighting conditions inside buildings.
• The datasets are highly useful to educate architectural and engineering students on façade and daylighting design. It also guides students to reproduce such lighting experiments and develop architectural configurations and prototypes addressing the lighting needs of occupants.
• The datasets can be used by architects, lighting specialists, interior designers, and building engineers who study the impact of architectural configurations and façades on indoor lighting conditions and individuals’ perceptions and circadian responses.
• The shared datasets could be used by computer vision researchers and machine learning specialists to train artificial intelligence for architectural applications and lighting-color interactions.
• The datasets could be used for perception studies using questionnaires to enquire about emotional responses to façade configurations, lighting conditions, and color rendering in architecture.

## 1 Data Description

**Fig. 1:**
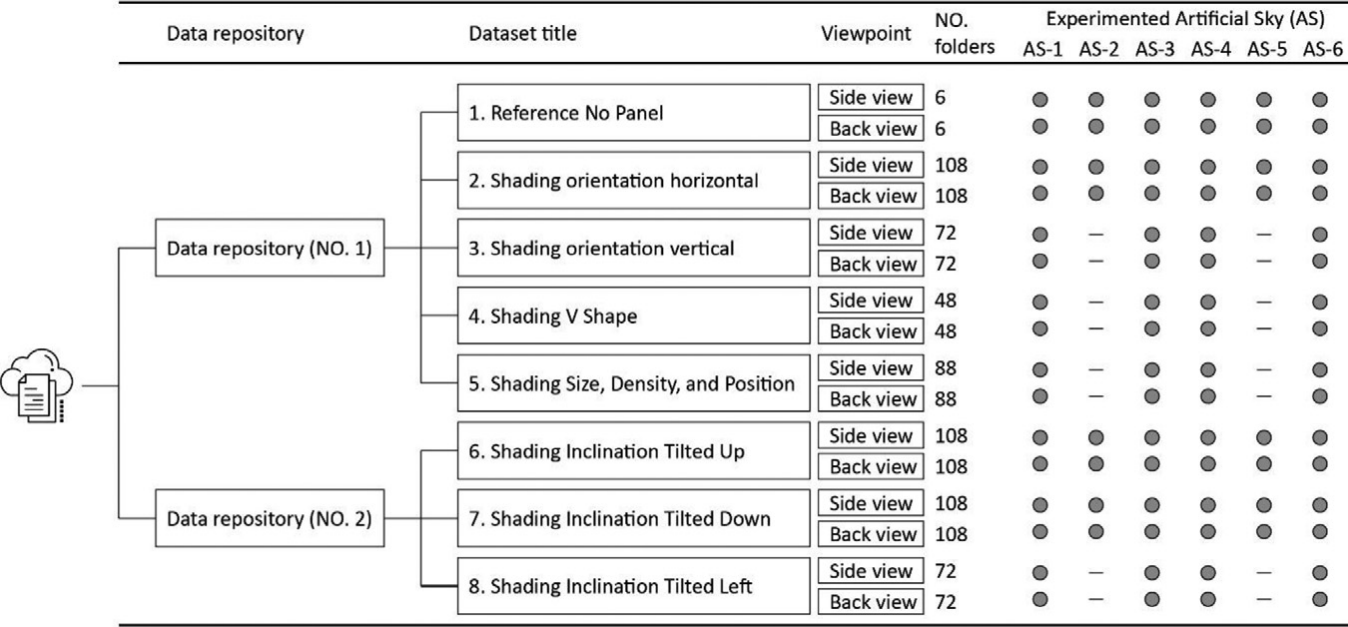
The data classification tree and the number of captured folders, and experimented lighting conditions in each Mendeley Data repository

Eight datasets, including 12000 images grouped in 1220 sets, are shared through two Mendeley Data repositories based on shading panel configurations. Fig. 1 displays the data classification tree and the number of captured folders and experimented lighting conditions shared in each dataset. Table 1 gives a brief description of each dataset. HDR images and their tone mapped plots, and false color maps of photobiological factors, i.e., photopic, melanopic, ratio of melanopic/photopic, and CCT units, are generated for side view captures of all datasets. The generated false color maps of side views are stored as a subfolder in each folder entitled ‘Analysis-Results’. Note that the saturated LDR images of all side view captures are cut and stored in a subfolder. LDR images of back view captures, however, have not been checked in terms of saturated images. An excel file entitled ‘Classifications' is provided in each dataset classifying the capture folders in terms of finishing exterior lighting conditions, colors, glossiness, and size, density, and position at the window where applicable. Tables 2 and 3 display the legend of acronyms and cell colors which are used in the Classification files. LDR images are provided in a JPG format. HDR images are generated with a *.hdr file extension. Tone mapped and false color plots are rendered in a PNG format. A single-frame plot of side views inside the model is also plotted for all datasets by using the tone mapped images. Four TXT files are added to repository No. 1 which contain the response function and photometric calibration coefficients of cameras used for side and back view captures.

**Table 1:** A brief description of each imagery dataset shared in the Mendeley Data repositories

Dataset Title	Dataset description
**Data Repository 1**	
1. Reference No Panel	• Present the base model which has no panel • Provide a baseline (ground truth) to detect impacts of shading panel configurations on lighting conditions inside the model
2. Shading orientation horizontal	• Present impacts of horizontal shading panels (with various colors and glossiness) on lighting conditions inside the model
3. Shading orientation vertical	• Present impacts of vertical shading panels (with various colors and glossiness) on lighting conditions inside the model
4. Shading V Shape	• Present impacts of V-shaped shading panel configurations (with various colors and glossiness) on lighting conditions inside the model
5. Shading Size, Density, and Position	• Present impacts of shading panels’ size, density, and position at the window (with matt blue colors) on lighting conditions inside the model
**Data Repository 2**	
6. Shading Inclination Tilted Up	• Present impacts of shading panels upwards inclination (with various colors and glossiness) on lighting conditions inside the model
7. Shading Inclination Tilted Down	• Present impacts of shading panels upwards inclination (with various colors and glossiness) on lighting conditions inside the model
8. Shading Inclination Tilted Left	• Present impacts of shading panels upwards inclination (with various colors and glossiness) on lighting conditions inside the model

**Table 2:** The legend of Acronyms used in Excel Classification files

Acronyms	Description
S	Saturated color (e.g., Sblue refers to saturated blue)
L	Light color (e.g., Lblue refers to light blue)
Cwhite	Cool white
Wwhite	Warm white
BlueR	Double-colored panels with saturated blue on top and saturated red at the bottom
RedB	Double-colored panels with saturated red on top and saturated blue at the bottom
Lblue-Yellow	Double-colored panels with light blue on top and saturated yellow at the bottom
Yellwo-Lblue	Double-colored panels with saturated yellow on top and light blue at the bottom
G	Glossy finishing
M	Matt finishing
H	Horizontal orientation
V	Vertical orientation
D	Density

**Table 3:** The legend of colors used in Excel Classification files

Cell colors	Description
	Folders containing captures of the case with saturated blue color panels
	Folders containing captures of the case with light blue color panels
	Folders containing captures of the case with saturated green color panels
	Folders containing captures of the case with light green color panels
	Folders containing captures of the case with saturated yellow color panels
	Folders containing captures of the case with saturated red color panels
	Folders containing captures of the case with double-colored panels

## 2 Experimental Setup, Architectural Configurations and Materials

**Fig. 2:**
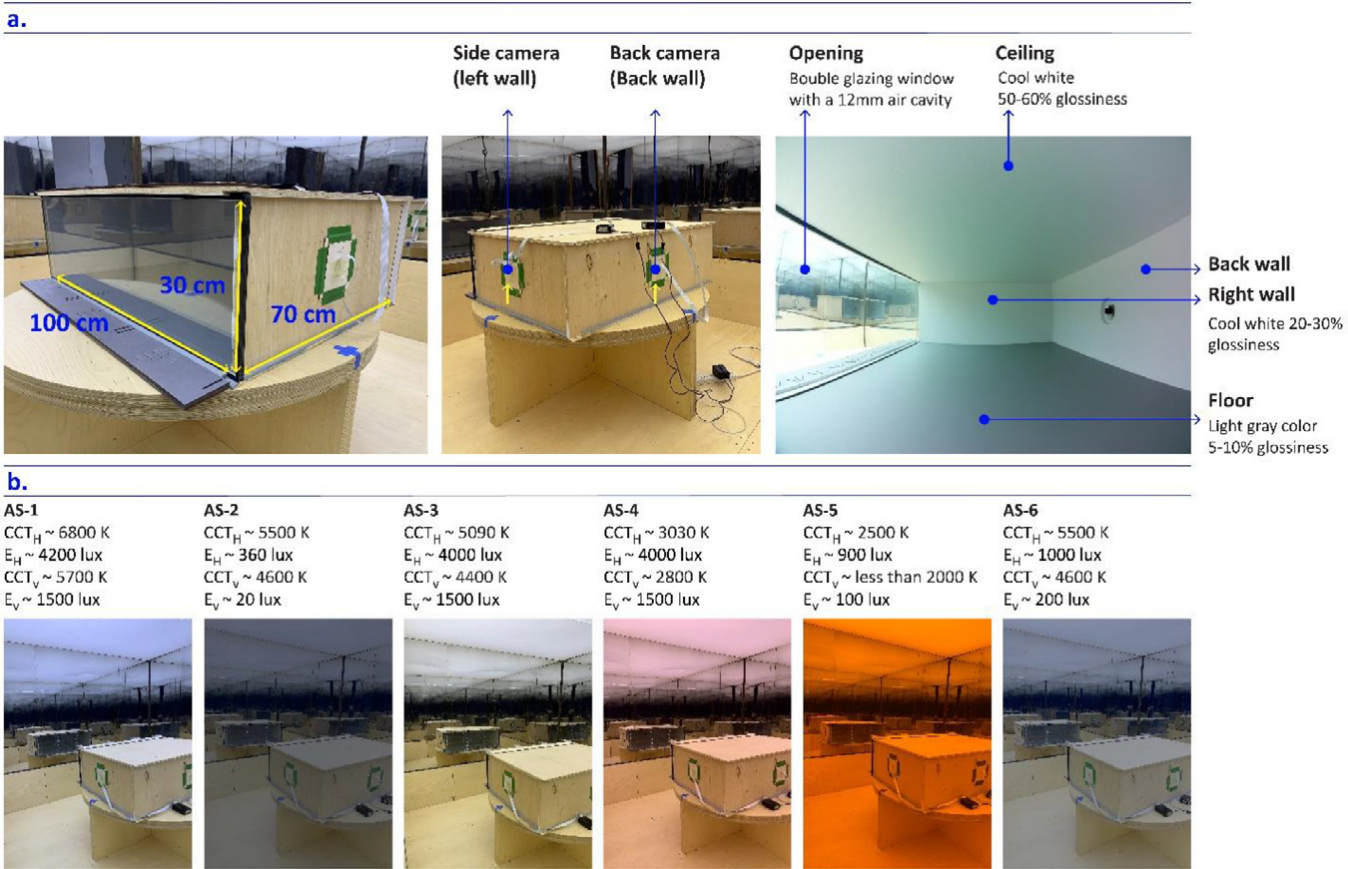
(a) The reference architectural model, which has no panel, mounted with Raspberry Pi cameras in the artificial sky chamber, (b) six lighting conditions used for the experiments.

The datasets are produced from experimental studies aimed at exploring impacts of shading panels’ configurations on lighting conditions in architecture. The overall experimental setup is presented in Fig. 2. The base model represents a generic space which can be used as an office, classroom, or cafeteria. The experiments were performed inside an artificial sky chamber simulating different exterior daylighting conditions. The artificial sky chamber is a human-scale mirror box equipped with a custom-made, tunable RGB light-emitting diode (LED) lighting system manufactured as Sunlike technology by Seoul Semiconductor [13]. The LED system is installed on the ceiling and covered with an acrylic diffuser. The dimensions and structure of the artificial sky is available by Groupe de recherche en ambiances physiques (GRAP [14]). Illuminance intensities (E) and CCTs of all experimental lighting conditions are measured at the horizontal (H) top surface and at the vertical (V) window surface of the model.

**Fig. 3:**
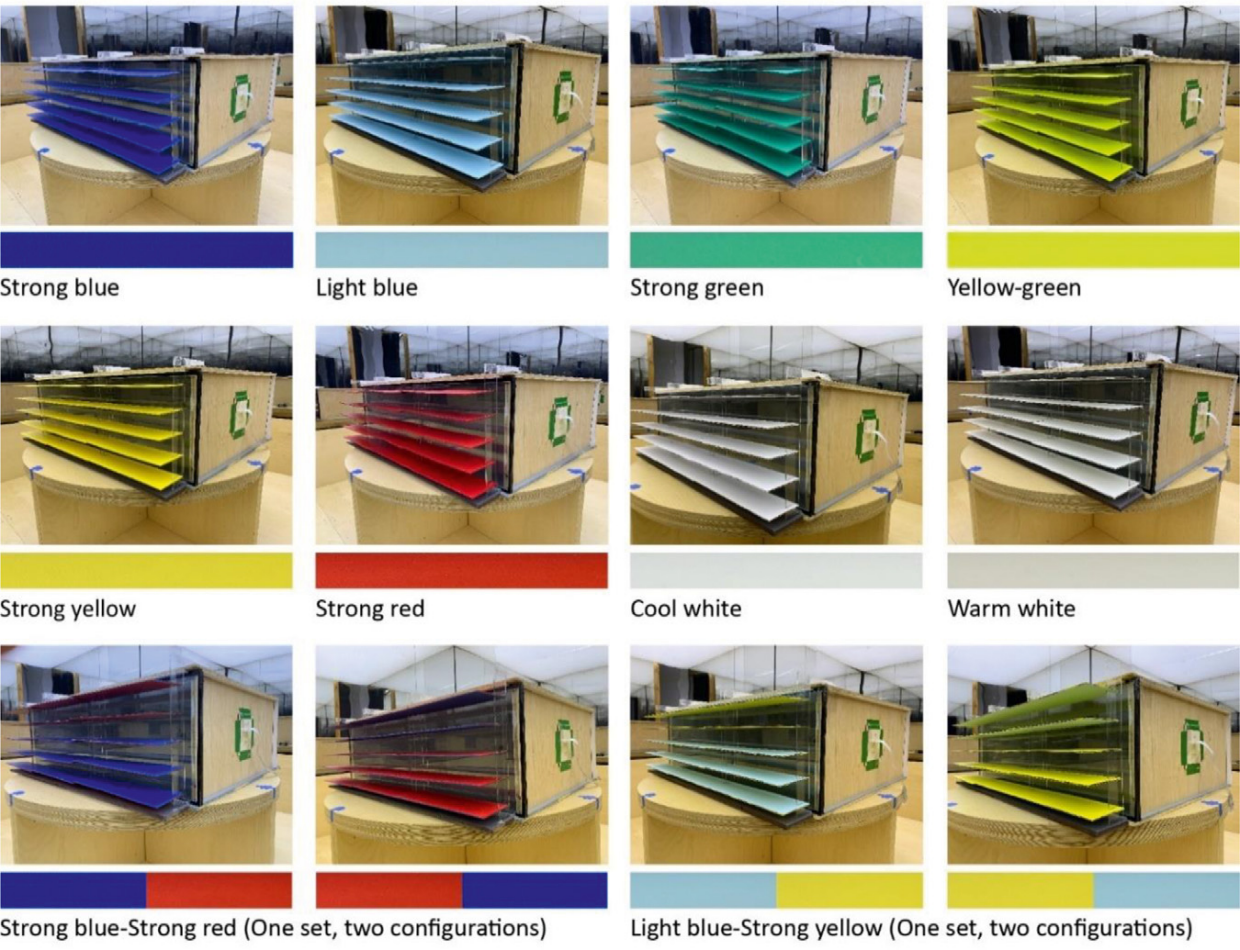
Colored shading panels produced with matt and glossy varnished finishing

**Fig. 4:**
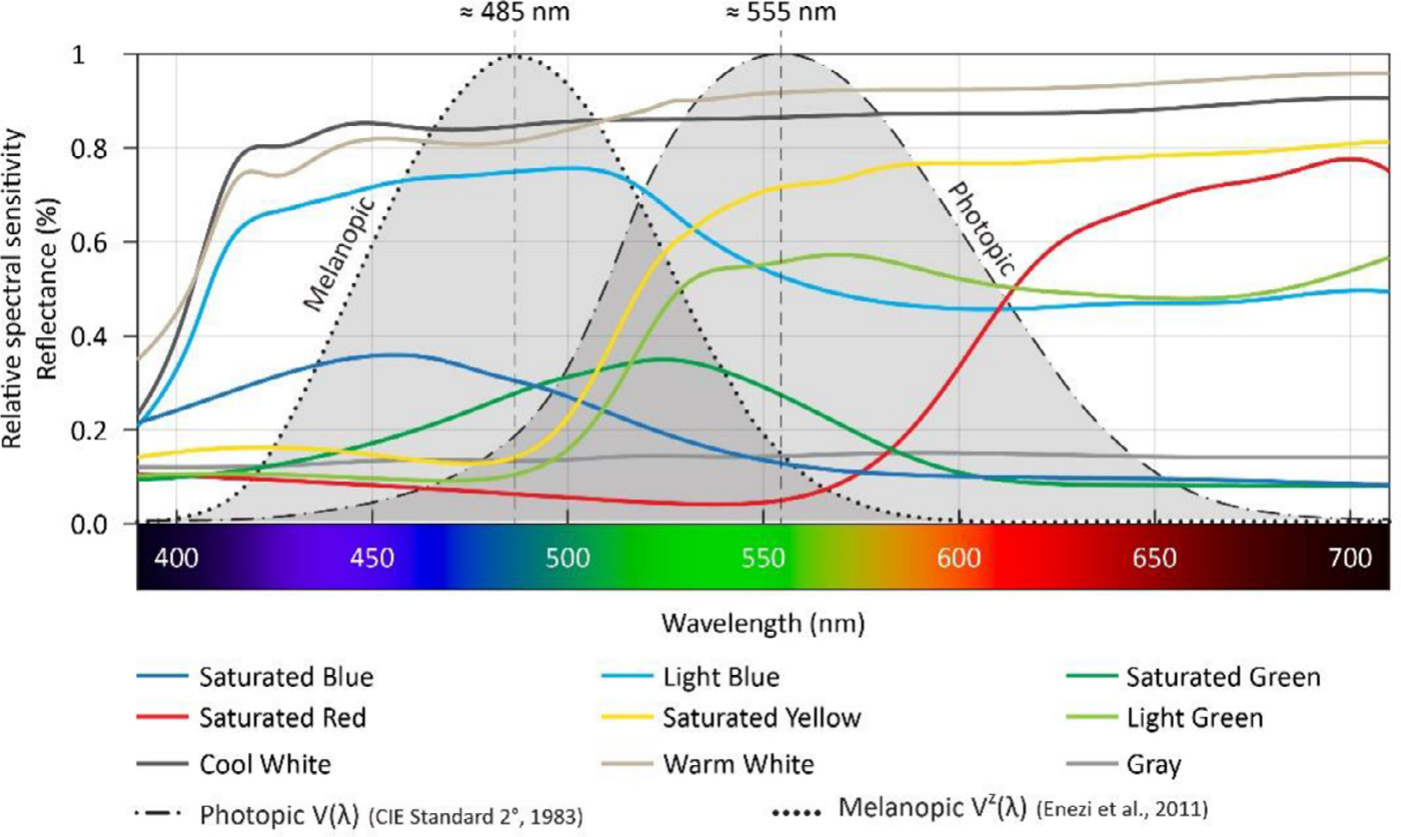
Relative spectral reflectance curves of matt-colored panels measured by Parsaee, et al. [1] and melanopic and photopic efficiency curves retrieved from Enezi, et al. [16], Amundadottir, et al. [17], DiLaura, et al. [18], CIE [19].

**Fig. 5:**
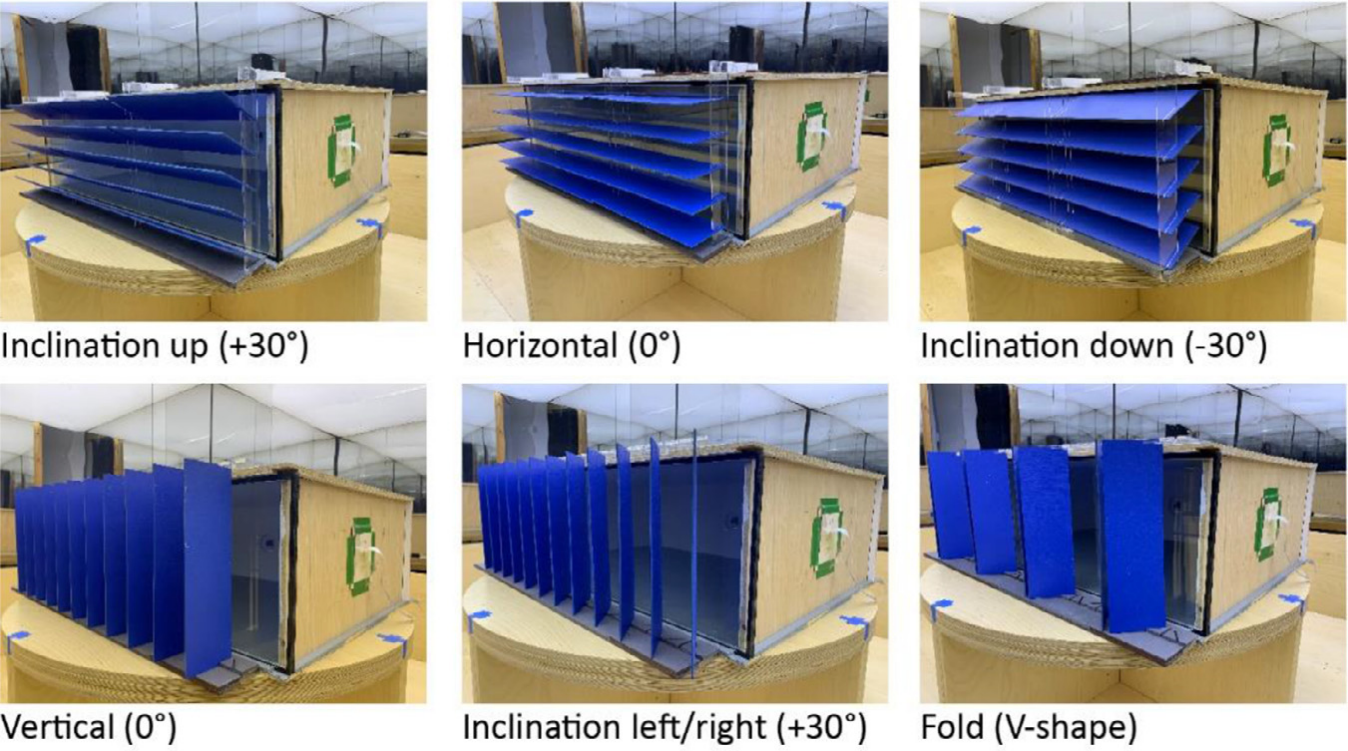
The experimented orientation, inclination, and shape of colored panels

**Fig. 6:**
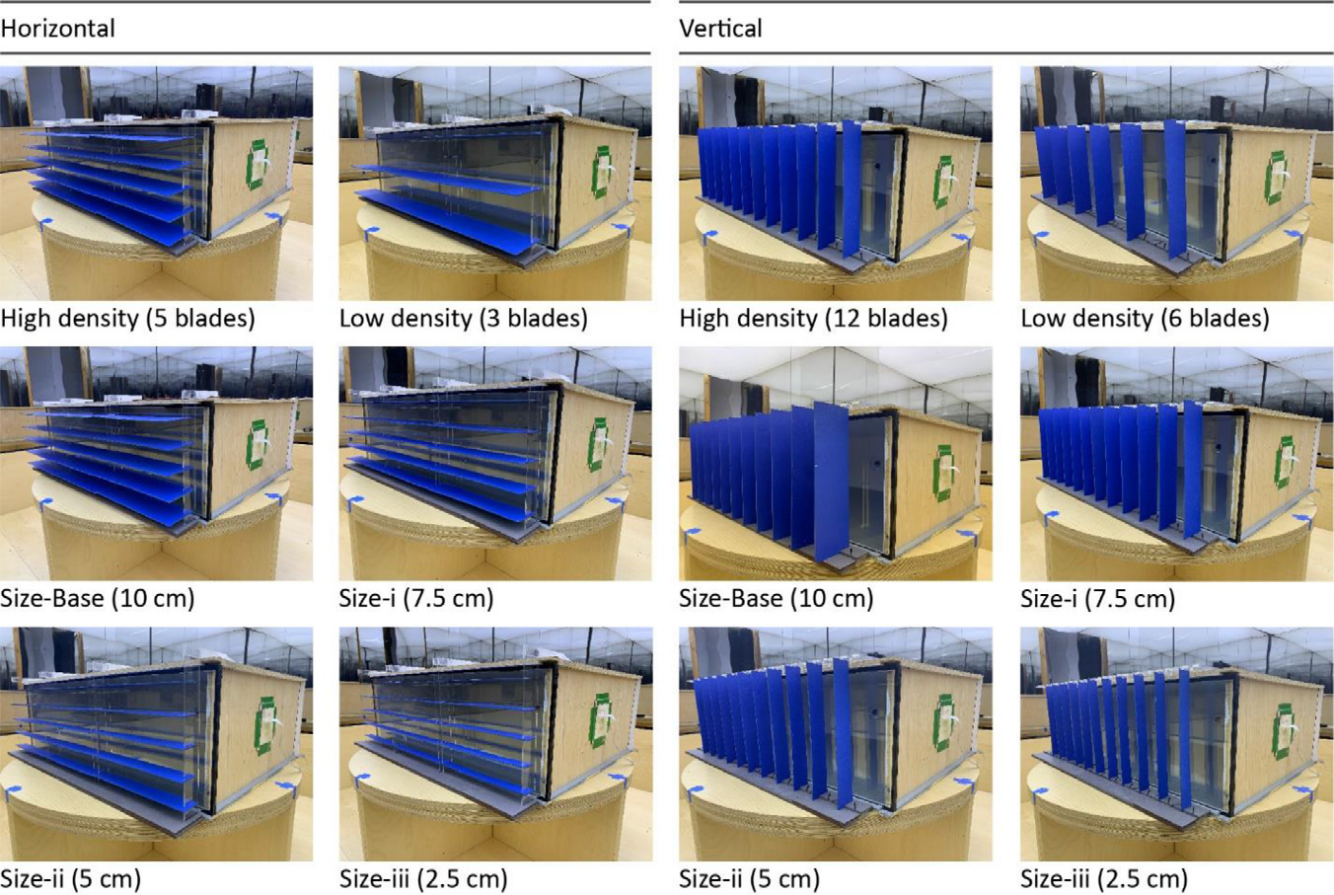
The density and size of saturated blue panels experimented in horizontal and vertical orientations

**Fig. 7:**
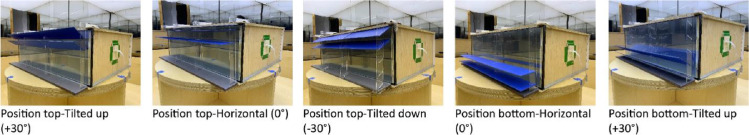
Panels’ positions at the window experimented for saturated blue colors with various inclinations

The base model and shading panels are produced by plywood at a 1:10 scale. The size and proportion of the base model represent a generic architectural space based on Jafarian, et al. [5,6], Poirier, et al. [7]. The model has three fully opaque sides and a 100% window made of a double-pane glazing with about 70-80% visual light transmission and a 12 mm air gap. All joints were completely sealed preventing light leakages as recommended by Ruck, et al. [8], Baker, et al. [9]. The interior walls of the model are painted in a cool white color with about 20% to 30% glossiness produced by SICO Evolution [15]. The ceiling of the model is also painted by the SICO Evolution [15] cool white color with 50% to 60% glossiness. A SICO Evolution [15] light-gray matt color with about 5% to 10% glossiness is used to paint the floor of the model. Twenty-three sets of shading panels were produced in different colors, glossiness, and sizes, as presented in Fig. 3. Eight sets of shading panels were painted and varnished with about 20-30% gloss in cool and warm white, saturated, and light blue, saturated green, light green, saturated yellow and saturated red colors. Two sets of panels were painted with double-colored sides with similar glossiness including saturated blue/saturated red and light-blue-saturated, yellow-colored sides. Ten shading panel sets with similar painted colors are also produced with matt finishing. Note that the double-colored sides panels offer two basic configurations in a horizontal orientation as shown in Fig. 3. Relative spectral curves of all matt-colored panels’ reflectance are measured by Parsaee, et al. [1] as depicted in Fig. 4. Colored shading panels were tested in horizontal and vertical orientations, upwards, downwards, and left/right inclinations, and folded in V-shape as illustrated in Fig. 5. Matt saturated blue colored panels are particularly built in four different sizes as shown in Fig. 6. These panels with multiple sizes were also experienced as low- and high-density installations at the window. The size and density variations of panels are tested in both horizontal and vertical orientations (refer to the dataset entitled ‘Shading Size, Density, and Position’). Matt saturated blue colored panels with typical sizes are experimented at top and bottom positions at the window with upwards, downwards, and horizontal inclinations, as displayed in Fig. 7.

Side and back view scenes inside the model are captured by two Raspberry Pi cameras installed in the middle of the side and back façades. Cameras were manufactured as RPi Camera-I by WaveShare [10]. RPi Camera-I is mounted with a fisheye lens offering about a 185-degree diagonal field of view. The camera has a fixed aperture of f/2 and a focal length of 1.55 mm. It has a 5-megapixel OV5647 sensor with a CCD size of 1/4 inch. The camera could capture images with 2592-by-1944-pixel resolutions. Further technical specifications of the camera and lens are provided by WaveShare [10].

## 3 Image Capturing and Post-Processing Procedures

The following steps explain the overall workflow conducted to capture LDR images and generate, calibrate and post-process HDR files.

1. **Capture bracketed images**: the Python program RaspiCamera [20] is developed to automatically capture bracketed LDR images via Raspberry Pi cameras. As shown in Fig. 8, the bracketed LDR images were captured from very dark to very bright high exposure values (i.e., around -2 EV to +3 EV) by changing shutter speeds from around 15 to 1/2 seconds. All LDR images were photographed with a fixed white balance (D65) and ISO of 100. LDR images are stored with a sRGB (i.e., standard Red, Green, and Blue) color space in a JPG format. Raspberry Pi's are both connected to the operator's laptop via remote desktop applications. The captured LDR images are then transferred to the laptop for post processing.

**Fig. 8:**
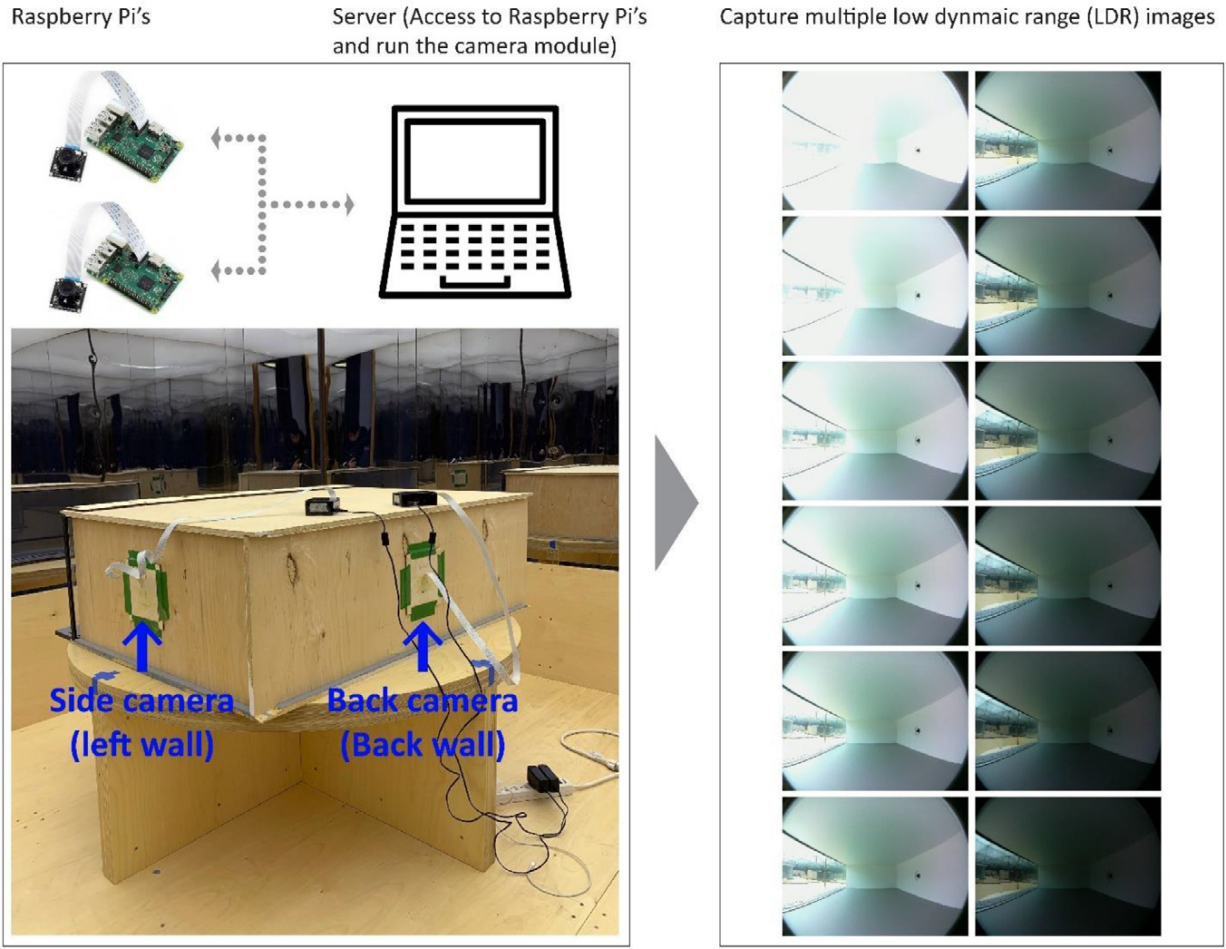
The workflow of capturing LDR image, generating calibrated HDR images
2. **Generate HDR images**: the Python program HDR Generator [21] is developed to produce HDR images by recovering camera response functions (CRFs) and merging LDRs. This python program uses OpenCV [22] and ExifTool [23] libraries to generate HDR images. As shown in Fig. 9, the CRFs are calculated for both cameras based on Debevec and Malik [24] which is available as an OpenCV method. The CRFs are shared alongside of the datasets. Note that the CRFs are generated for the first reference model captures (under lighting conditions of AS-1) and subsequently used to produce all other HDR images. LDR images are also cropped to cut the black regions of fisheye captures. Over and under saturated LDR images are manually discarded as recommended by Debevec and Malik [24], Pierson, et al. [25]. HDR images are stored with a *.hdr file extension.

**Fig. 9:**
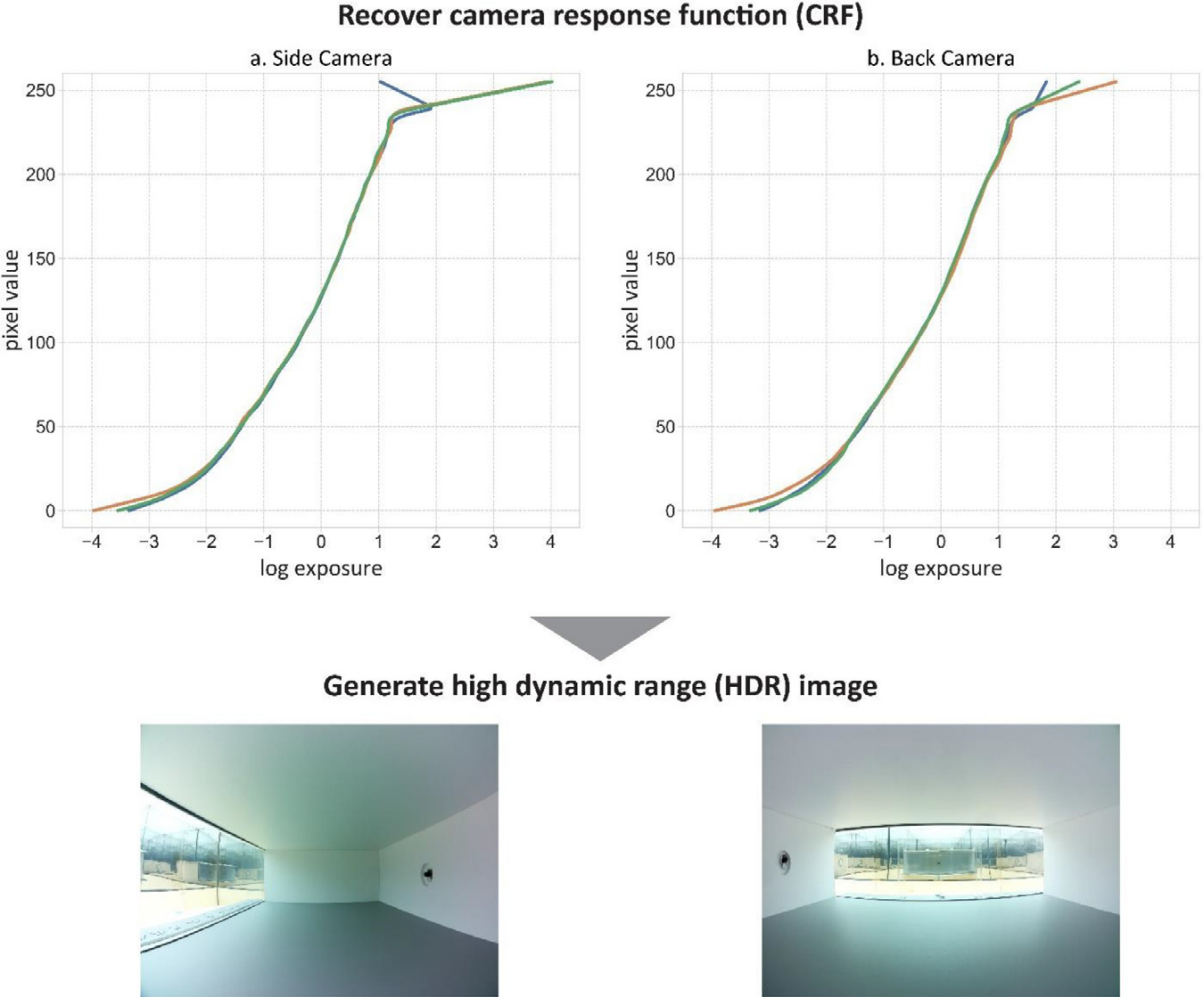
Generate HDR images by recovering response curves for side and back view cameras based on Debevec and Malik [24] offered by OpenCV [22].
3. **Calibrate color channels of HDR images**: RGB and XYZ (CIE tristimulus) channels of HDR images are calibrated for both cameras to enable accurate photometric and photobiological studies. Protocols for chromaticity calibration of cameras are fully explained by Jung [11], Jung and Inanici [12]. In brief, photometric and chromaticity properties, i.e., Illuminance and CIE-XYZ, of multiple scenes are recorded by using a calibrated Konica Minolta CL-200A photometer [26]. Note that the illuminance intensity is equal to CIE-Y. HDR images of the scenes are also simultaneously generated by both cameras. Photometric and chromaticity properties of all HDR images generated by each camera are calculated based on equations 1-3 provided by Jung [11], Jung and Inanici [12]. More specifically, equation (1) converts CIE-XYZ to RGB values. Equation (1) enables deriving RGB values of the photometer measurements of XYZ. Equation (2) enables RGB to CIE-XYZ conversions. Equation (2) is used to calculate XYZ values from the RGB properties of HDR images. The calibration coefficients of RGB and XYZ channels are then derived by fitting the calculated photometric and chromaticity properties of the HDR images to the absolute measured values recorded by the chroma meter as shown in equation (3). Note that the averages of RGB and CIE-XYZ values of all pixels in an HDR image are used to determine the calibration coefficients. The Python program HDRI Photobiological Visualizer [27] is developed to facilitate photometric and chromaticity calculations of HDR images. Fig. 10 shows the fitness curves plotted for both cameras.

**Fig. 10:**
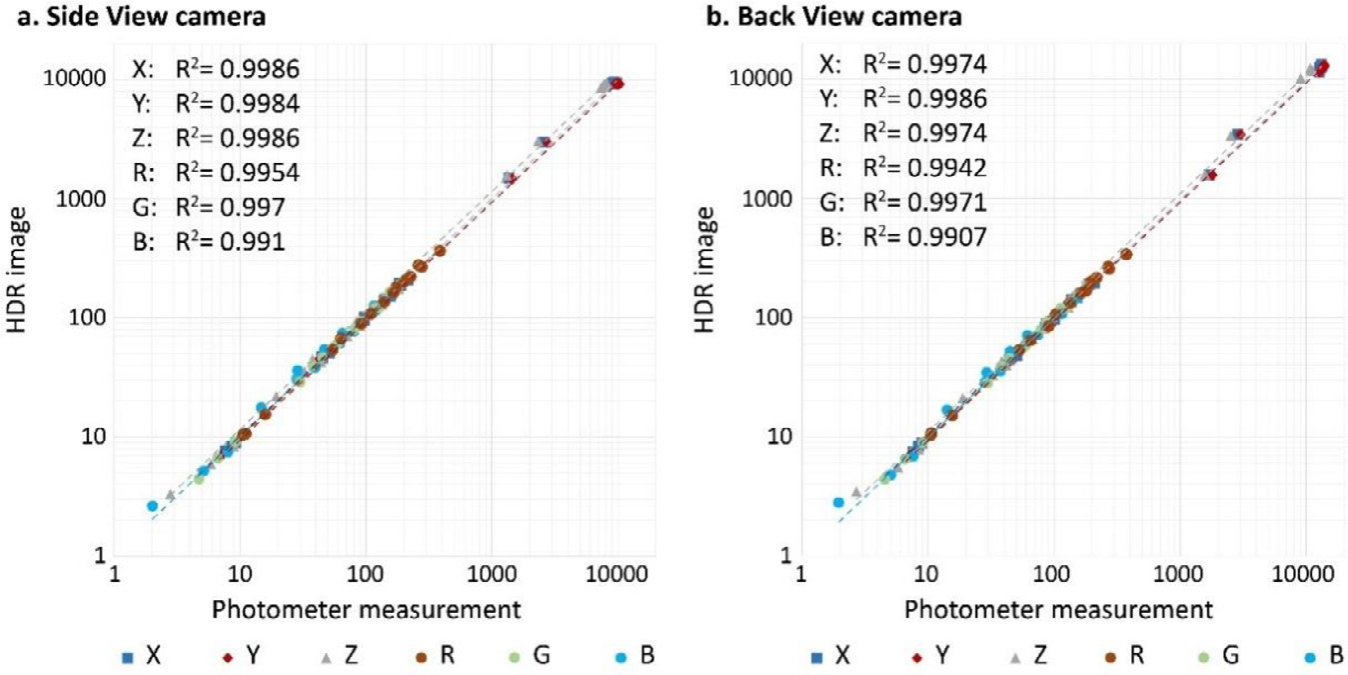
Photometric and chromaticity calibrations of HDR images captured by side and back cameras based Jung [11], Jung and Inanici [12].

CIE–XYZ conversion to sRGB

(1) $$\begin{array}{c} \left[\begin{array}{c} R\\ G\\ B \end{array}\right]=\left[\begin{array}{ccc} 3.2406 & -1.5372 & -0.4986\\ -0.9689 & 1.8758 & 0.0415\\ 0.0557 & -0.2040 & 1.0570 \end{array}\right]\left[\begin{array}{c} X\\ Y\\ Z \end{array}\right] \end{array}$$

sRGB conversion to CIE–XYZ

(2) $$\left[\begin{array}{c} X\\ Y\\ Z \end{array}\right]=\left[\begin{array}{ccc} 0.4124 & 0.3576 & 0.1805\\ 0.2127 & 0.7152 & 0.0722\\ 0.0193 & 0.1192 & 0.9505 \end{array}\right]\left[\begin{array}{c} R\\ G\\ B \end{array}\right]$$

Calibration factors to fit the calculated values from HDR images to photometer meauremnets

(3) $$\left\{ \begin{array}{c} X_{photometer}=Average\;\left(X_{HDRI\;pixels}\right)*C_{X}\\ Y_{photometer}=Average\;\left(Y_{HDRI\;pixels}\right)*C_{Y}\\ \begin{array}{c} Z_{photometer}=Average\;\left(Z_{HDRI\;pixels}\right)*C_{Z}\\ R_{photometer}=Average\;\left(R_{HDRI\;pixels}\right)*C_{R}\\ G_{photometer}=Average\;\left(G_{HDRI\;pixels}\right)*C_{G}\\ B_{photometer}=Average\;\left(B_{HDRI\;pixels}\right)*C_{B} \end{array} \end{array}\right.$$

(3.1) $$\begin{array}{c} \mathrm{for}\;\mathrm{the}\;\mathrm{side}\;\mathrm{came}\mathrm{ra}\left\{ \begin{array}{c} C_{X}=7.044325\\ C_{Y}=\;6.450126\\ C_{Z}=5.081117\\ C_{R}=\;0.98\\ C_{G}=1.008\\ C_{B}=\;1.05 \end{array}\right. \end{array}$$

(3.2) $$\begin{array}{c} \mathrm{for}\;\mathrm{the}\;\mathrm{back}\;\mathrm{camera}\left\{ \begin{array}{c} C_{X}=7.493178\\ C_{Y}=\;6.9805\\ C_{Z}=5.474306\\ C_{R}=\;0.96\\ C_{G}=1.001\\ C_{B}=\;1.025 \end{array}\right. \end{array}$$

**Fig. 11:**
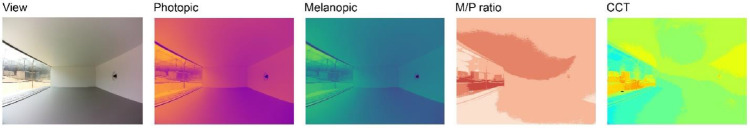
An example of false color maps rendered for a side view.

4. **Compute photobiological units and render false color maps**: Equations 3-6 and the chromaticity calibration coefficients calculated for each camera are used to render false color maps of photobiological parameters, i.e., photopic, melanopic, ratio of melanopic/photopic M/P, and CCT units. More specifically, equations 4 and 5 compute photopic (in $cd/m^{2}$) and melanopic (in $EMcd/m^{2}$) units based on Jung [11], Jung and Inanici [12]. Equation (6) enables CCT calculations based on the McCamy [28] method for the CIE-D65 white point as used for the sRBG color space. The Python program HDRI Photobiological Visualizer [27] enables rendering such false color maps of photobiological units. Fig. 11 illustrates an example of false color maps rendered for a side view.

Photopic Luminance in cd/m^2^

(4) $$\left(0.2127\;*R_{pixel-i}*C_{R}+\;0.7152*\;G_{pixel-i}*C_{G}+0.0722*B_{pixel-i}*C_{B}\right)$$

Equivalent melanopic Luminance in EMcd/m^2^

(5) $$\left(0.0013*R_{pixel-i}*C_{R}+\;0.3812*\;G_{pixel-i}*C_{G}+0.6175*B_{pixel-i}*C_{B}\right)$$

CCT calcualtions for CIE–D65 white point

(6) $$CCT=449*n^{3}+3525*n^{2}+6823.3*n+5518.87$$

where

(6.1) $$x=\frac{X}{X+Y+Z}$$

(6.2) $$y=\frac{Y}{X+Y+Z}$$

(6.3) $$n=\frac{x-0.3320}{0.1858-y}$$

The described methodology could also be used for similar computational lighting analysis employing HDR imagery and post-processing techniques. The methodology is simplified to be fully understandable for architects and designers. The developed Python programs also enable batch processing to compute and render false color maps of large imagery datasets.

## Ethics Statements

The presented datasets do not involve any human subjects, or animal experiments, or using social media platforms.

## CRediT Author Statement

**Mojtaba Parsaee:** Conceptualization, Methodology, Software, Data curation, Formal analysis, Validation, Visualization, Investigation, Writing - Original draft preparation; **Claude MH Demers:** Supervision, Funding acquisition, Writing - Reviewing and Editing; **Marc Hébert:** Supervision, Funding acquisition, Writing - Reviewing and Editing; **Jean-François Lalonde:** Supervision, Funding acquisition, Writing - Reviewing and Editing.

## Declaration of Competing Interest

The authors declare that they have no known competing financial interests or personal relationships that could have appeared to influence the work reported in this paper.

## Data Availability

Imagery datasets for photobiological lighting analysis of architectural models with shading panels (No. 1). Imagery datasets for photobiological lighting analysis of architectural models with shading panels (No. 2) (Original data) (Mendeley Data).
